# Density-Dependent Regulation of Brook Trout Population Dynamics along a Core-Periphery Distribution Gradient in a Central Appalachian Watershed

**DOI:** 10.1371/journal.pone.0091673

**Published:** 2014-03-11

**Authors:** Brock M. Huntsman, J. Todd Petty

**Affiliations:** Division of Forestry and Natural Resources, West Virginia University, Morgantown, West Virginia, United States of America; North Carolina State University, United States of America

## Abstract

Spatial population models predict strong density-dependence and relatively stable population dynamics near the core of a species' distribution with increasing variance and importance of density-independent processes operating towards the population periphery. Using a 10-year data set and an information-theoretic approach, we tested a series of candidate models considering density-dependent and density-independent controls on brook trout population dynamics across a core-periphery distribution gradient within a central Appalachian watershed. We sampled seven sub-populations with study sites ranging in drainage area from 1.3–60 km^2^ and long-term average densities ranging from 0.335–0.006 trout/m. Modeled response variables included per capita population growth rate of young-of-the-year, adult, and total brook trout. We also quantified a stock-recruitment relationship for the headwater population and coefficients of variability in mean trout density for all sub-populations over time. Density-dependent regulation was prevalent throughout the study area regardless of stream size. However, density-independent temperature models carried substantial weight and likely reflect the effect of year-to-year variability in water temperature on trout dispersal between cold tributaries and warm main stems. Estimated adult carrying capacities decreased exponentially with increasing stream size from 0.24 trout/m in headwaters to 0.005 trout/m in the main stem. Finally, temporal variance in brook trout population size was lowest in the high-density headwater population, tended to peak in mid-sized streams and declined slightly in the largest streams with the lowest densities. Our results provide support for the hypothesis that local density-dependent processes have a strong control on brook trout dynamics across the entire distribution gradient. However, the mechanisms of regulation likely shift from competition for limited food and space in headwater streams to competition for thermal refugia in larger main stems. It also is likely that source-sink dynamics and dispersal from small headwater habitats may partially influence brook trout population dynamics in the main stem.

## Introduction

Historical debate over population regulation focused on the relative occurrence of density-dependent (DD) vs. density-independent (DI) mechanisms (*see* reviews by Murdoch [1], Krebs [2], and Hixon et al. [3]). Currently it is accepted that both DD and DI processes interact to affect the dynamics of most natural populations [4]–[6]. For example, Prevateli et al. [7] showed that measures of both density and precipitation best explained the dynamics of two rodent species. Both DD and DI mechanisms have been shown to influence population dynamics of mammals [7], [8], birds [9], [10], amphibians [11], [12], and fishes [6], [13]–[15], providing evidence for the prevalence of both mechanisms in influencing population dynamics. Consequently, ecologists recognize that it is not only important to consider how DD and DI forces interact, but also how the prevalence of these factors may vary across the landscape [16], [17].

A promising approach to understanding complex population dynamics across heterogeneous landscapes is to apply a core-periphery perspective [9], [17], [18]–[20]. A population living within its core distribution is considered to be at a location where it is least susceptible to environmental variability due to habitat specific adaptation. A population at its periphery is more susceptible to those environmental characteristics and therefore less adapted to the local habitat [17]. As a consequence, it is expected that a population should be at its highest densities at the core and decrease with distance from the core [16]. Populations within the core of their distribution are then expected to be strongly regulated by local DD mechanisms, such as competition [17]. This occurs not only because densities are high near the core, but also because variability in important environmental factors should be low. At the periphery of the population distribution, highly variable environmental conditions and relatively low population densities are expected to result in weak DD regulation and increasing importance of local DI factors on population dynamics [17]. Successful applications of core-periphery concepts to explain landscape scale variability in population dynamics have been made in terrestrial, terrestrial/aquatic [9], [18], [21]–[24], and aquatic environments (see Nicola et al. [6], Haak et al. [20], Kim & Lapointe [25]).

River networks, or riverscapes [26], provide a unique opportunity to study the complexities of population dynamics at a scale relevant to aquatic metapopulations [25], [27]–[29]. Watersheds often show strong environmental gradients over relatively small spatial scales. For example, tributary-main stem confluences can support highly diverse habitat due to transitioning between large and small stream dynamics over short flow distances [30], [31]. Gradients in environmental conditions such as temperature are particularly important for cold-water specialists like salmonids that require specific thermal ranges for optimal performance within a habitat [32]–[34]. Petty et al. [28] observed high mobility in brook trout exposed to elevated thermal conditions within a large main stem habitat, while tributary residents showed less mobility, likely due to relatively lower thermal stress. Additionally, demographic rates of fish species (e.g., survival and birth rates) have been shown to differ based on whether fish were present in their core or peripheral distribution within the same watershed [27]. Therefore, applying a core-periphery approach within a riverscape can potentially give an important perspective of population dynamics that could not be obtained by focusing on one location.

Understanding the strength of DD vs. DI mechanisms limiting fishes across population distribution gradients is crucial given current climate change scenarios. Numerous studies recognize that alterations to climatic variables could have substantial effects on fish species distributions [20], [35]–[37]. However, impacts may also be observed affecting fish productivity [38]. This could be especially detrimental to populations supplementing their productivity through exploiting peripheral habitat patches. For example, substantial gains in productivity have been shown for stream fishes able to access highly productive floodplain habitat [29], [39]. However, higher temperatures and more sporadic flooding events could substantially reduce access to these supplementary feeding habitats or increase mortality through fish stranding. For cold-water species with distinct core-periphery distributions (*i.e*. brook trout, [27]), the strength of climatic variables in limiting population productivity may be strongly linked to location within their spatial distribution. In order to properly assess how such populations would respond to climate change predictions, we must understand the relative importance of DD vs. DI factors along a species distribution gradient. Therefore, our objectives for this study were to: 1- quantify the relative importance of DD and DI controls on brook trout population dynamics, 2- quantify a stock-recruitment relationship for brook trout populations within a known source headwater stream; and 3- quantify temporal variation in brook trout densities and spatial variation in brook trout carrying capacities across a core-periphery distribution gradient.

### Study Area and Expectations

**Figure 1:**
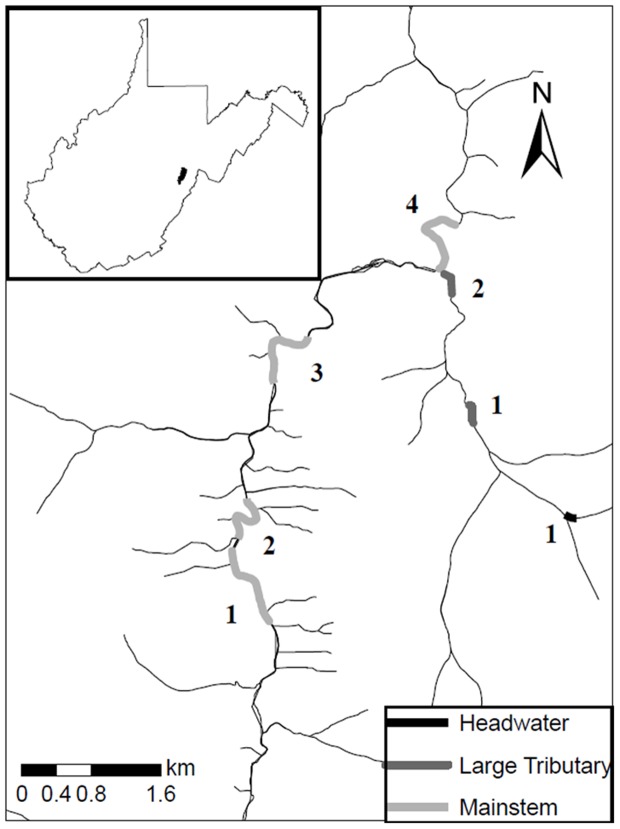
Seven study sites within the upper Shavers Fork watershed in Pocahontas and Randolph counties, WV.

The upper Shavers Fork is a large (i.e., >150 km^2^ basin area), high elevation (originates at 1500 m) watershed located in the central Appalachian Mountains of eastern West Virginia (Pocahontas and Randolph Counties, Figure 1). The Shavers Fork is part of the Cheat River drainage flowing north to its confluence with the Monongahela River. A detailed description of the Shavers Fork can be found in Petty et al. [27].

Brook trout inhabit a broad range of stream sizes in the Shavers Fork watershed, ranging from extremely small headwater seeps (<1 km^2^) to large main stem reaches (>100 km^2^). The main stem is relatively wide and shallow, has a low gradient and an open canopy, is warmer and more productive, and possesses a more diverse brook trout prey assemblage than smaller tributaries [28]. Greater than 80% of brook trout reproduction occurs in headwater streams with drainage areas less than 3 km^2^, and brook trout reproduction has rarely been observed in streams with drainage areas greater than 15 km^2^ [27]. As a consequence, brook trout density is highest in small headwater streams (core habitat) and decreases with increasing stream size down to only a few individuals in larger main stem sites (periphery habitat) (Table 1).

**Table 1:** Site description for each stream selected for this study.

	Headwater	Large Tributary		Main Stem			
	1	1	2	1	2	3	4
DA (km^2^)	1.3	10.4	15.4	27.9	30.3	41.1	59.8
Elevation (m)	1255	1184	1149	1177	1170	1157	1137
Stream length (m)	150	306	315	944	863	919	1007
Max July temp (°C)	14.8 (0.2)	14.1 (0.2)	15.9 (0.2)	19.7 (0.4)	19.7 (0.3)	20.0 (0.3)	19.5 (0.5)
Prey density (#/m^2^)	1834	1019	721	NA	3917	2363	1580
Total brook trout density (#/m)	0.335 (0.040)	0.132 (0.036)	0.081 (0.012)	0.023 (0.006)	0.014 (0.004)	0.022 (0.004)	0.006 (0.001)
Brook trout density C.V.	36.28	81.86	44.53	83.81	91.69	59.14	61.44
Brook trout L.A. density (#/m)	0.103 (0.010)	0.063 (0.014)	0.039 (0.005)	0.013 (0.003)	0.007 (0.002)	0.017 (0.003)	0.004 (0.001)
Brook trout S.A. density (#/m)	0.154 (0.029)	0.039 (0.011)	0.034 (0.008)	0.007 (0.003)	0.004 (0.002)	0.004 (0.001)	0.001 (0.001)
Brook trout YOY density (#/m)	0.077 (0.023)	0.030 (0.023)	0.008 (0.004)	0.003 (0.002)	0.003 (0.002)	0.001 (0.001)	0.001 (0.000)
Mean brook trout SL (mm)	96.5 (1.7)	114.3 (2.5)	117.4 (2.5)	133.2 (7.1)	133.3 (10.3)	148.3 (7.0)	128.3 (16.1)
Brown trout density (#/m)	0	0	0.001 (0.001)	0.011 (0.003)	0.014 (0.002)	0.007 (0.001)	0.007 (0.002)
Rainbow trout density (#/m)	0	0.001 (0.001)	0.013 (0.005)	0.018 (0.005)	0.011 (0.003)	0.015 (0.003)	0.002 (0.001)
Fish richness	1	5	13	NA	17	17	NA
Spawning intensity	22	3	2	0	0	0	0

Mean density (s.e.) of total brook trout (SAFO), as well as large adult (L.A.), small adult (S.A.), and young-of-the-year (YOY). Similarly, brown trout, and rainbow trout densities are reported as means captured from 2002–2011 by site. Mean brook trout standard length (SL) is reported for just adults. Coefficient of variation (C.V.) was also estimated for total brook trout densities. Max July temp is the mean daily maximum temperature for the month of July averaged over the 10 year study period. Prey density values are estimates from unpublished data of benthic kick-net samples for the 2011 spring sampling season. Fish richness values represent the number of different fish species captured from each location (J.T. Petty *unpublished data*). Spawning intensity is the total number of redd counts observed during the Fall spawning season [27]. Drainage area is represented by DA.

Based on what we know about brook trout populations in this watershed, we expected the following results: 1- local DD mechanisms should be the dominant control on brook trout dynamics within headwater (i.e., core) habitats and decline in importance with movement towards larger main stem (i.e., periphery) habitats; 2- local DI mechanisms should be the strongest factor limiting brook trout population dynamics within larger, periphery habitats; and 3- temporal variation in population densities should be highest at the periphery and lowest in the core.

## Materials and Methods

### Study Sites

Seven sites within the upper Shavers Fork watershed were chosen for this study (Figure 1, Table 1). These sites were selected to fully represent potential habitat available for brook trout within this watershed. Headwater site 1 has a drainage area less than 3 km^2^. Large tributary 1 and 2 have drainage areas greater than 3 km^2^, but have been shown to support low levels of brook trout spawning (Table 1, [27]). The final four main stem sites (1, 2, 3, and 4) all have drainage areas outside of what has been shown to support spawning activity (>16 km^2^, [27]). All stream lengths were established in 2001, and the same length of stream was monitored on subsequent sampling events. Stream lengths were standardized by stream width, where a site's length was 40 times mean stream width and at least 150 m long.

### Trout Population Sampling

Every spring from 2002 until 2011 (end of May-Early June), brook trout, brown trout (*Salmo trutta*), and rainbow trout (*Oncorhynchus mykiss*) were sampled from the 7 study sites and measured for standard length (SL) and mass (g). Trout abundances were converted to densities by dividing abundance by reach specific lengths (#/m stream length). Trout were collected with backpack electrofishing units (Smith-Root, DC, 60 Hz. 400–600 V) in an upstream direction using a single pass procedure [40].

Single pass electrofishing makes it possible to sample large areas over time, which was a priority for us. However, this approach can produce biased abundance estimates if there is significant spatial or temporal variation in first pass capture efficiencies [40]. In order to quantify site-to-site and year-to-year variation in capture probabilities among study sites, three-pass depletion methods were used at all sites in 2002 and 2006 and at the headwater 1 and main stem 2 sampling sites in 2011. During this sampling, block nets were placed at the beginning and end of each reach, all trout were captured and removed on each pass, and we used the analytical methods of Hense et al. [40] to quantify brook trout capture probabilities at each site and each sampling event. Capture probabilities ranged from 0.75–0.78, which was consistent with previous studies in the region [27], [40]. There were no consistent patterns of sample bias at any given site, nor in any given year. Instead, spatio-temporal variation of ±3% appeared to represent a range of random variation associated with sampling error. Given these findings, we assumed a temporally and spatially constant sampling efficiency and did not apply correction factors across study sites or years.

### Ethics Statement

After measurements were taken, fish were placed in a live-well until they recovered from electrofishing. All sampling was approved by the committee of animal use and care (IACUC) of West Virginia University (most recent protocol number 11–0507).

### Water Temperature and Flow

Stream temperature was collected from all study sites using HOBO Water Temp Pro V2 data loggers from 2002–2011. Average maximum stream temperature for July and average maximum daily stream temperature from April-June (growing period for brook trout, [41]) were temperature indices used for DI models. Discharge data were downloaded from a local U.S. Geological Survey stream gaging station at the Cheat bridge (USGS 03067510). Estimates of mean discharge (*Q*) from March-June were used as a DI mechanism. This is approximately when brook trout emerge from eggs [42], and has been shown to be a time during a salmonid's life cycle when they are highly susceptible to discharge events [43].

### Statistical Analyses

We constructed general linear models within the R statistical program (R Development Core Team 2011) to test for density-dependent (DD) and density-independent (DI) controls on brook trout population dynamics [14], [44], [45]. Specific response variables analyzed were brook trout per capita rate of change for young-of-the-year (YOY), adult, and total brook trout (*r* = ln(*n_t_*/*n_t−1_*)). Length-frequency histograms from Petty et al. [27] were used to differentiate small adult, large adult, and YOY brook trout size classes. Based on this criterion, YOY were defined as individuals smaller than or equal to 60 mm SL, small adults were anything between 60 and 100 mm SL, and large adults were anything greater than or equal to 100 mm SL at the time of sampling. Although the YOY size class represents a true representation of age and denotes trout known to have been produced the previous fall, the small and large adult size classes likely do not represent a true distinction of age [27]. As such, all analyses for adult size classes represent the combination of both small and large adult size classes, but does not represent true age class or sexual maturity.

The candidate set of models for each response variable was evaluated using an information-theoretic approach and Akaike Information Criterion corrected for small sample size (AICc, [46]). This approach ranks a suite of candidate models by maximum parsimony. Within this approach, AIC_c_ weights (*w_i_*) were constructed for each candidate model to evaluate the strength of the best model compared to the rest of the candidate set. Criteria for model interpretation were similar to that utilized by Grossman et al. [14], [44]. The model with the highest *w_i_* was compared to each model in the candidate set by dividing the best model by each candidate model. This percentage then gave an estimate of the relative strength of the best model over the remaining models in the candidate set. Only models with *w_i_* values greater than 10% of the best model's *w_i_* were considered interpretable models (*see* Table S1 for all constructed models) [14], [44], [46].

Candidate sets of models were constructed to be similar among sites, in order to compare the strength of DD vs DI, and to identify local temporal brook trout dynamics. Due to extremely low competitor densities in tributary sites (especially headwater 1 and large tributary 1, see Table 1), competitor densities were not included in candidate sets of models. Therefore, post-hoc analyses were conducted on main stem sites to explore the potential effects of competitors on brook trout population dynamics. We used correlation analysis (pearson's correlation) between total competitor densities (*i.e*. rainbow and brown trout) and brook trout densities to test for temporal autocorrelation between the time-series at each periphery site.

Additionally, pearson's correlation coefficient was used to test for spatial correlation in the time series of brook trout densities among all sites. Regression analysis was then used to test for relationships between differences in stream sizes and brook trout densities between sites. We also used regression analysis to test for relationships between swim distance and brook trout densities as a means of exploring the potential effects of dispersal among sites on local population dynamics.

Since adult brook trout were consistently found throughout the watershed, but only a few YOY were found in larger main stem habitat (Table 1), adult brook trout carrying capacities were estimated for each site. To estimate carrying capacity, adult brook trout per capita rate of change was plotted as a function of adult brook trout densities. The x-intercept then represents the stable equilibrium point for the adult brook trout population at each site and the theoretical adult brook trout carrying capacity [47].

Stock recruitment assessment was addressed in the headwater 1 site using multiple stepwise linear regression analysis. YOY densities were modeled as a function of similar predictor variables outlined for YOY per capita rate of change, with a few exceptions. No YOY density predictor variables were included in the analysis, and adult brook trout densities at time *t-1* and time *t* were included.

To test whether variability in brook trout densities increased with distance from the core on the riverscape, coefficients of variation (C.V.) were estimated from brook trout time series for each site. These values were then plotted against site drainage area, to determine if variability in density increased with drainage area.

## Results

A total of 1737 brook trout was captured over the course of this study with a maximum observed in 2005 (*n* = 344) and a minimum in 2010 (*n* = 80). No rainbow trout were captured in headwater 1, and no brown trout were captured in headwater 1 or large tributary 1 (Table 1). The highest brown trout densities were observed at main stem 2, and rainbow trout were most dense at main stem 1 (Table 1). Mean brook trout density was greatest at headwater 1 and decreased with an increase in drainage area (Table 1). In the core (headwater 1), small adult densities were highest, followed by large adult and YOY density (Table 1). For all other sites, large adult densities were highest, followed by small adults and YOY (large tributary and main stem, Table 1).

**Figure 2:**
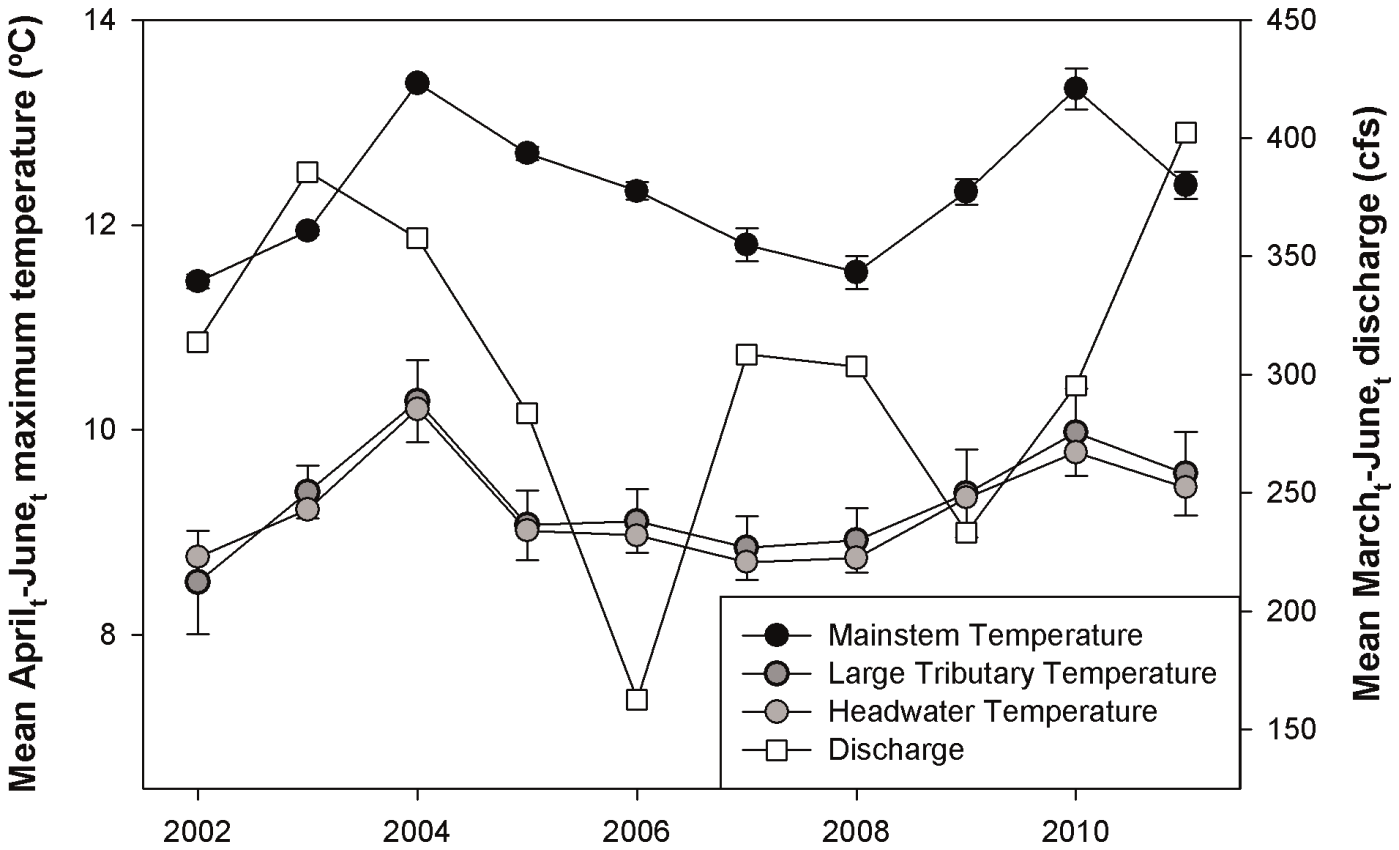
Time series plot of mean temperature and discharge across site types. Time series plots are of year versus mean April_t_-June_t_ maximum water temperature and mean March_t_-June_t_ discharge (Q). Means were for the 2 large tributary sites and the 4 main stem sites.

**Figure 3:**
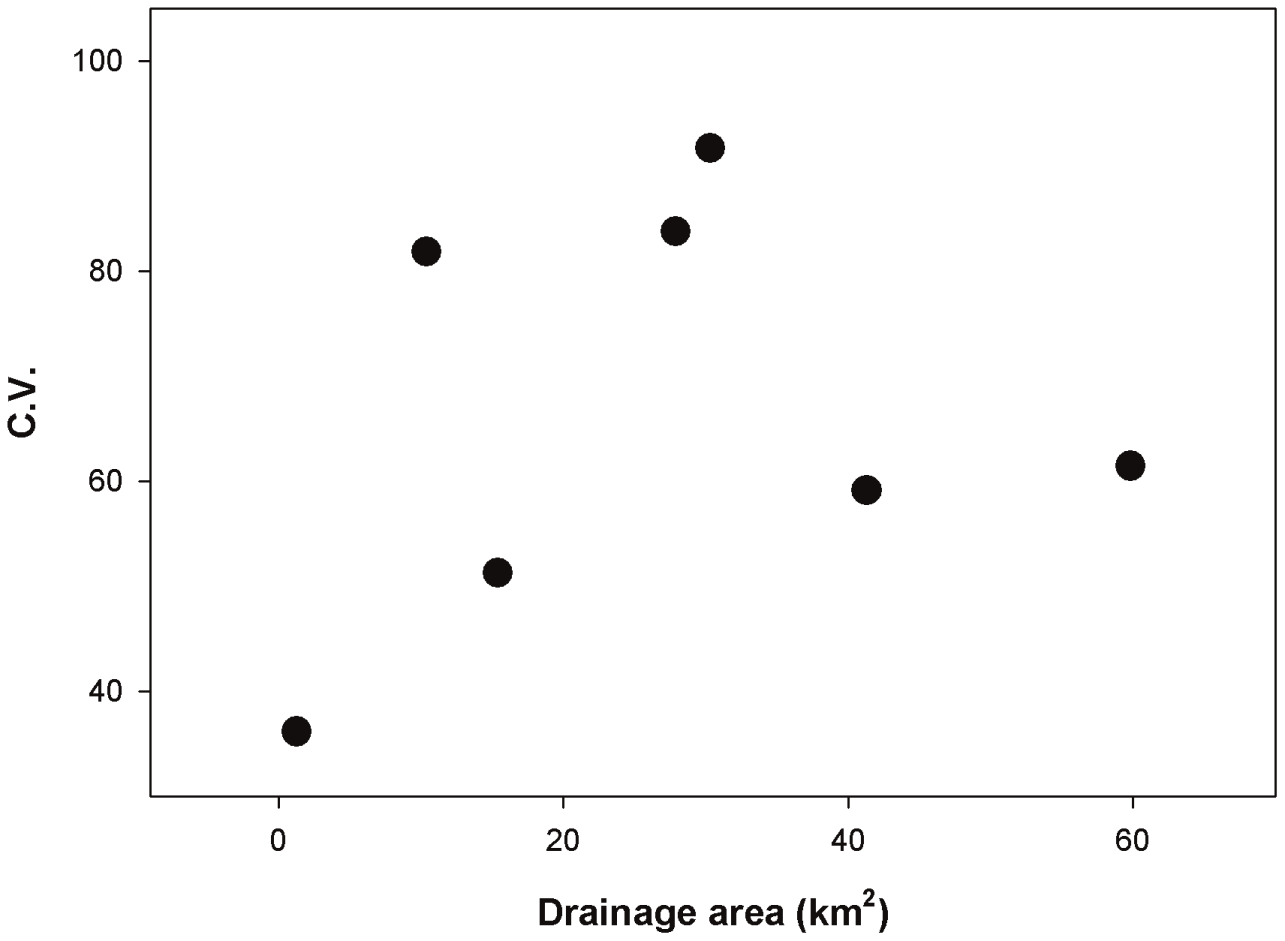
Brook trout density coefficient of variation (C.V.) as a function of stream drainage area.

Brook trout densities as well as environmental characteristics (temperature and flow) varied considerably over time (Figures 2 and 3). Temporal variance in brook trout density demonstrated a hump-shaped relationship with drainage area (Figure 3). Coefficient of variation (C.V.) in brook trout density was highest in streams with drainage areas ranging from 10–30 km^2^ (Figure 3). C.V. of brook trout density was considerably lower in the smallest headwater habitat and also declined in the two largest sites (Figure 3). The observed increase in population variance from small to intermediate sized streams was consistent with expectations. However, the decline in C.V. in the two largest sites was unexpected.

### Total per capita growth rate

**Figure 4:**
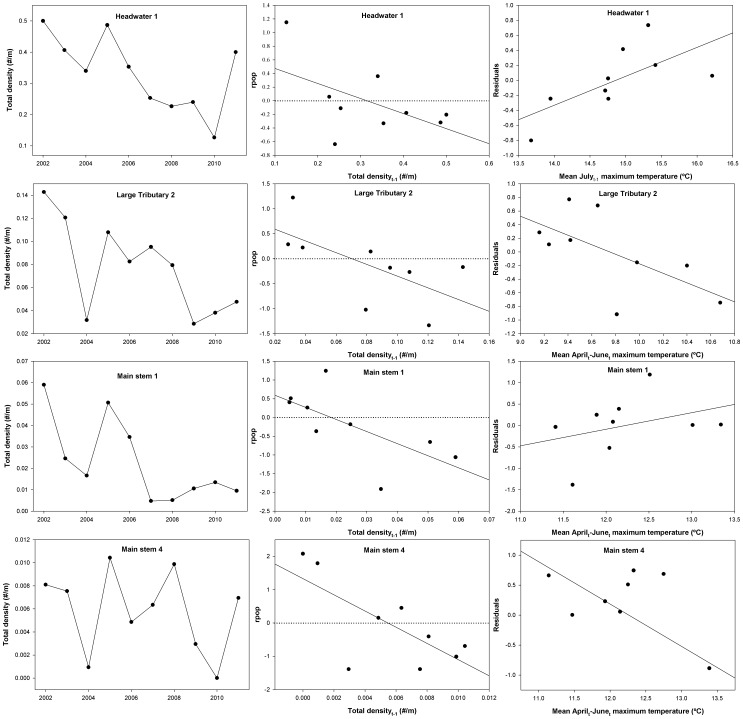
Time series, best model, and residual plots for total brook trout analyses. Plots of total brook trout density time series, the highest Akaike weighted (*w_i_*) model, and the residuals of the best model as a function of the best DI predictor variable for per capita rate of change in the total brook trout population (rpop). The residual plot was selected based on the highest *R^2^* model in the multi-mechanism set that also contained the highest weighted predictor variable. Horizontal dotted lines represent the local carrying capacity for each site.

A total of 13 candidate models was constructed for total brook trout per capita growth (Table S2). In five of the seven sites analyzed, DD models (trout density_t−1_) were the most parsimonious for total per capita growth, whereas a complex model (both DD and DI) best explained variability in the headwater 1 site (Figure 4, Table 2). The only site with a DI model as the highest ranked in the candidate set was in the main stem 2 site, where per capita growth was positively correlated with April-June temperature. In 4 of 7 sites, a substantial amount of variability was explained by multi-mechanism models, involving combined effects of spring or summer water temperature and trout density the previous year (*R^2^* in Table 2).

**Table 2:** Results from candidate models using AIC_c_ for the 7 study sites.

Res.	Predictor	Headwater			Large Tributary						Main Stem											
Var.	Variable	1			1			2			1			2			3			4		
		*w_i_*	R^2^	dir.	*w_i_*	R^2^	dir.	*w_i_*	R^2^	dir.	*w_i_*	R^2^	dir.	*w_i_*	R^2^	dir.	*w_i_*	R^2^	dir.	*w_i_*	R^2^	dir.
rpop	dtrout_t−1_	0.227*	0.28	-	0.491*	**0.29**	-	0.357*	**0.22**	-	0.709*	**0.46**	-	0.253*	0.16	-	0.591*	**0.38**	-	0.710*	**0.50**	-
	dyoy_t-1_	0.073*	0.08	-				0.147*	0.05	-							0.073*	0.01	-			
	su_t-1_T	0.076*	0.09	+	0.139*	0.06	+	0.126*	0.01	+	0.068	0.09	-	0.122*	0.01	-				0.031	0.00	+
	sp_t_T	0.051*	0.00	+	0.109*	0.01	+	0.132*	0.02	-	0.077*	0.12	+	0.464*	**0.27**	+	0.211*	0.22	+	0.041	0.07	-
	sp_t_Q	0.113*	0.16	+	0.108*	0.01	+	0.177*	0.09	+	0.054	0.04	-	0.118*	0.01	+	0.073*	0.01	-	0.049	0.10	+
	su_t-1_T+dtrout_t-1_	0.429*	0.72	+/−	0.123*	0.57	+/−	0.010	0.22	+/−	0.025	0.49	+/−	0.007	0.17	+/−				0.042	0.58	+/−
	sp_t_T+dtrout_t-1_	0.006	0.29	-/-	0.013	0.29	-/-	0.014	0.28	-/-	0.035	0.53	+/−	0.017	0.31	+/−	0.021	0.42	+/−	0.106*	0.66	-/-
radult	dadult_t-1_	0.589*	**0.64**	-	0.420*	**0.31**	-	0.488*	**0.32**	-	0.697*	**0.46**	-	0.246*	0.14	-	0.622*	**0.40**	-	0.682*	**0.52**	-
	dyoy_t-1_	0.008	0.05	-				0.094*	0.01	-							0.064*	0.00	-	0.036	0.08	-
	su_t-1_T	0.006	0.00	+	0.318*	0.26	+	0.129*	0.08	+	0.066	0.09	-	0.125*	0.00	-				0.026	0.01	+
	sp_t_T	0.007	0.02	+	0.091*	0.28	+	0.093*	0.01	-	0.085*	0.14	+	0.460*	**0.25**	+	0.192*	0.22	+	0.035	0.07	-
	sp_t_Q	0.010	0.09	+	0.082*	0.01	+	0.127*	0.08	+	0.003	0.05	-	0.127*	0.00	+	0.070*	0.02	-	0.040	0.10	+
	su_t-1_T+dadult_t-1_	0.343*	0.82	+/−	0.062*	0.52	+/−	0.014	0.33	+/−	0.022	0.48	+/−	0.008	0.16	+−/				0.042	0.60	+/−
	sp_t_T+dadult_t-1_	0.017	0.64	-/-	0.013	0.33	+/−	0.014	0.33	-/-	0.050	0.57	+/−	0.015	0.28	+−/	0.021	0.43	+/−	0.114*	0.68	-/-
ryoy	dadult_t-1_	0.088*	0.11	+	0.209*	0.07	-	0.023	0.20	+												
	dyoy_t-1_	0.288*	**0.31**	-	0.422*	**0.20**	-	0.737*	**0.63**	-												
	su_t-1_T	0.235*	0.28	+				0.013	0.09	+												
	sp_t_T	0.055*	0.01	-	0.155*	0.00	+	0.030	0.25	-												
	sp_t_Q	0.145*	0.20	+	0.164*	0.02	+	0.008	0.00	-												
	su_t-1_T+dyoy_t-1_	0.114*	0.62	+/−				0.126*	0.75	+/−												

Only interpretable predictor variables in at least one site are provided (see Table S2 for all models analyzed). The first value represents the Akaike's weight (*w_i_*) given to each model in the candidate set followed by the *R^2^* statistic and the direction of the relationship. Bold values represent the best model in each candidate set. Abbreviations are as follows: rpop  =  per capita growth rate (r = ln(n_t_/n_t−1_) for the total brook trout population, radult  = r for adults, ryoy  = r for young-of-the-year, dtrout  =  density of all brook trout, dadult  =  density of adult brook trout, dyoy  =  density of young-of-the-year brook trout, sp_t_T  =  mean April-June maximum temperature, su_t-1_T  =  mean July maximum temperature, and sp_t_Q  =  mean March-June discharge. Missing values represent predictor variables that were correlated with another predictor variable in the candidate set and therefore removed. No models were constructed for ryoy at main stem sites because few YOY were found in those sites. Models with an * were considered interpretable models using criteria from Grossman et al. [14].

### Adult per capita growth rate

**Figure 5:**
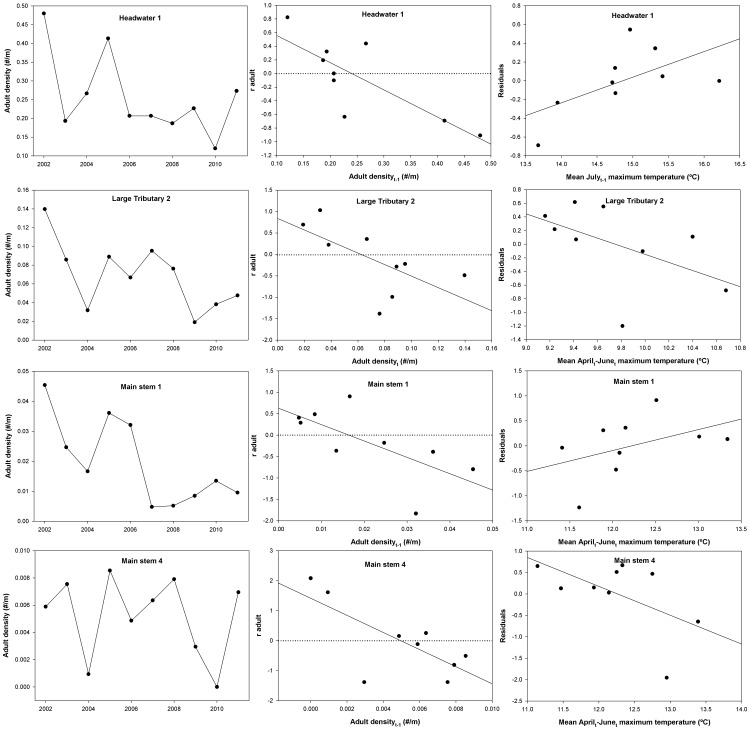
Time series, best model, and residual plots for adult brook trout analyses. Plots of adult brook trout density time series, the highest Akaike weighted (*w_i_*) model, and the residuals of the best model as a function of the best DI predictor variable for per capita rate of change in the adult brook trout population (radult). The residual plot was selected based on the highest *R^2^* model in the multi-mechanism set that also contained the highest weighted predictor variable. Horizontal dotted lines represent the local carrying capacity for each site.

A total of 13 models was also constructed for adult brook trout per capita growth (Table S2). Adult brook trout per capita growth was best explained by a DD model in six of the seven sites (adult density), with adult population growth rates in main stem 2 being positively correlated to April-June stream temperature (Table 2, Figure 5). As expected, a large amount of variability in adult *r* was explained by the best model in the headwater site (*R^2^* = 0.64, Table 2), although the best models in the large tributaries explained less variability than most other sites (*R^2^* = 0.31 and 0.32, Table 2). Surprisingly, a large amount of variability (*R^2^* = 0.46–0.52, Table 2) was explained by the best models (DD) in the peripheral sites (main stem 1, 3, and 4). Multi-mechanism models that included water temperature and density effects were interpretable in 2 of 3 smaller sites (headwater 1 and large tributary 1), whereas only one of four sites in the periphery had an interpretable multi-mechanism model (Table 2, Figure 5).

### YOY per capita growth rate

**Figure 6:**
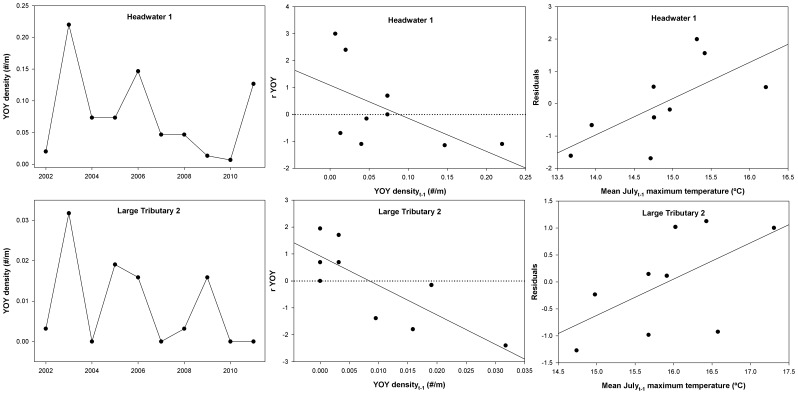
Time series, best model, and residual plots for young-of-the-year brook trout analyses. Plots of YOY brook trout density time series, the highest Akaike weighted (*w_i_*) model, and the residuals of the best model as a function of the best DI predictor variable for per capita rate of change in the YOY brook trout population (ryoy). The residual plot was selected based on the highest *R^2^* model in the multi-mechanism set that also contained the highest weighted predictor variable. Horizontal dotted lines represent the local carrying capacity for each site.

**Figure 7:**
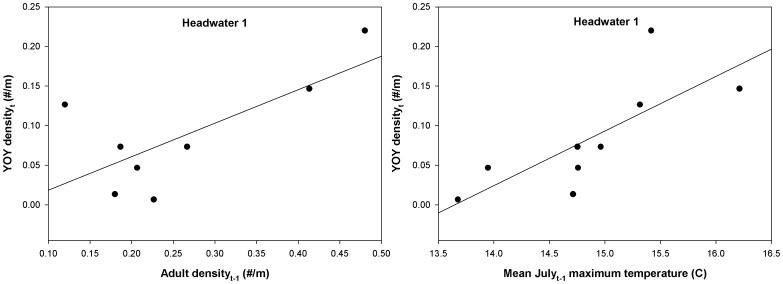
Predictors of young-of-the-year recruitment in the core. Stock-recruitment curve for YOY brook trout density as a function of (A) adult brook trout density the previous year and (B) mean July maximum temperature at the headwater 1 site.

YOY per capita growth was analyzed for headwater 1 and the two large tributary sites only due to few YOY being found in peripheral sites over the course of the study. All three sites analyzed showed DD being the most parsimonious model, where per capita growth of YOY was negatively correlated with YOY densities the previous year (Table 2). The same multi-mechanism model was interpretable for both headwater 1 and large tributary 2 sites and included a positive effect of July temperature and a negative effect of YOY density on YOY per capita growth (Table 2, Figure 6). Multiple stepwise regression revealed that July temperature, adult densities, and March-June discharge all were important variables influencing YOY brook trout densities within the core headwater 1 site (Table 3, *R^2^* = 0.96, *p*<0.001). The positive relationship between adult densities and YOY densities the following year indicates a significant stock-recruitment relationship within the core site (Figure 7A) that is also significantly modified by July water temperatures (Table 3, Figure 7B).

**Table 3:** Multiple stepwise regression predicting density of YOY at Headwater 1.

Variable	Estimate	P-value	Partial R^2^	Final model R^2^	Final model p-value
intercept	−0.9751	0.001		0.96	0.0006
dadult_t-1_	0.2956	0.005	0.38		
su_t-1_T	0.0561	0.002	0.44		
sp_t_Q	0.0005	0.002	0.14		

The final regression equation is y = 0.2956dadult_t-1_ + 0.0561su_t-1_T + 0.0005sp_t_Q −0.9751.

### Correlation between brook trout and competitors

Total brook trout densities showed strong positive temporal correlation with densities of potential competitors within three of four peripheral sites (*Pearson's r*; 0.81, 0.72, 0.80 for main stem 1, 2, and 3 respectively). Brook trout and competitor densities were not significantly correlated in the largest main stem site (*r* = 0.06).

### Pairwise correlation among sites

**Figure 8:**
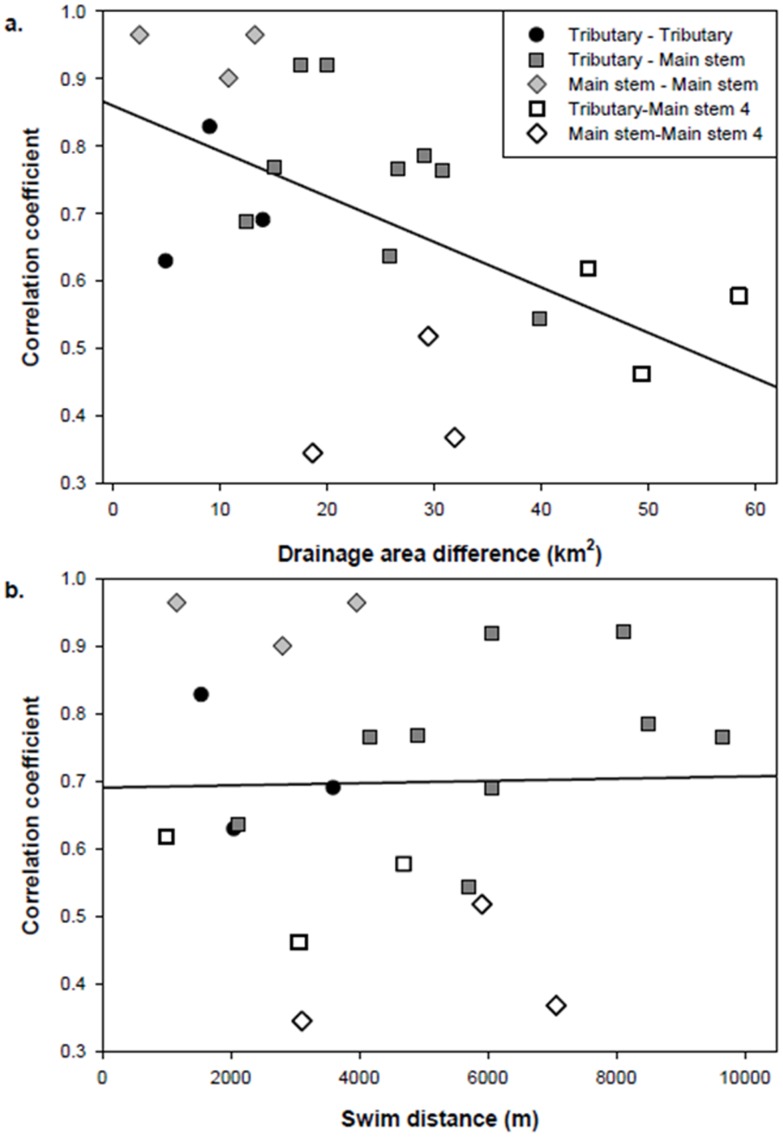
Brook trout pairwise correlation analysis among sites. a) Correlation of brook trout densities regressed against drainage area differences between sites (*p* = 0.01, R^2^ = 0.27) and b) correlation of brook trout densities regressed against swim distances between sites (*p* = 0.93, R^2^ = 0.00).

We observed high temporal correlation in the brook trout time series for most pairwise site comparisons (Figure 8). There was a significant decrease in pairwise correlation between sites as the difference in drainage area between the sites increased (Figure 8a, *R^2^* = 0.27, *p* = 0.01). However, the main stem 4 site appeared to be out of phase with other similar sized main stem sites (Figure 8). Similar analyses relating correlations in brook trout densities between sites close in proximity (swim distance) did not show a significant relationship (Figure 8b, *R^2^*<0.01, *p* = 0.93) between the strength of correlation and swim distance between the sites. For example, high positive year-to-year correlations in brook trout density was observed between sites separated by as much as 5–10 km swim distance (Figure 8b). Again, many of the lowest pairwise correlations involved comparisons with the main stem 4 site.

## Discussion

Density-dependent regulation was prominent throughout the watershed. The finding that population dynamics within the core headwater habitat was strongly regulated by density-dependent mechanisms was expected and consistent with previous studies [9], [44], [45], [48], [49]. High population densities along with species adaptation to environmental conditions characteristic of the core habitat are expected to result in strong density-dependent regulating mechanisms around a relatively stable carrying capacity [17]. The high brook trout density relative to variability (C.V., Table 1) and prevalence of density-dependence suggests the core headwater habitat conforms to these expectations.

Surprisingly, brook trout dynamics within more peripheral habitats downstream also were strongly regulated by density-dependent mechanisms. We expected brook trout populations to become increasingly influenced by density-independent factors, such as water temperature and flow, with distance away from core habitats. Previous studies outside of a species core distribution have found density-independent mechanisms to be the dominant factor limiting populations [6], [50]–[52]. However, we found that density-dependence was a dominant regulating force on brook trout, regardless of spatial distribution within the watershed. Density-dependence has also been shown to regulate brook trout at broader spatial scales, from Michigan streams [45], [53] to streams in North Carolina [44]. To our knowledge, however, our study is the first to document density-dependent regulation of brook trout populations both within small headwater streams and within larger streams that represent the periphery of their distribution. Consequently, our results coupled with previous studies provide evidence for how extensive density-dependent regulation is for brook trout populations in the Appalachians.

**Figure 9:**
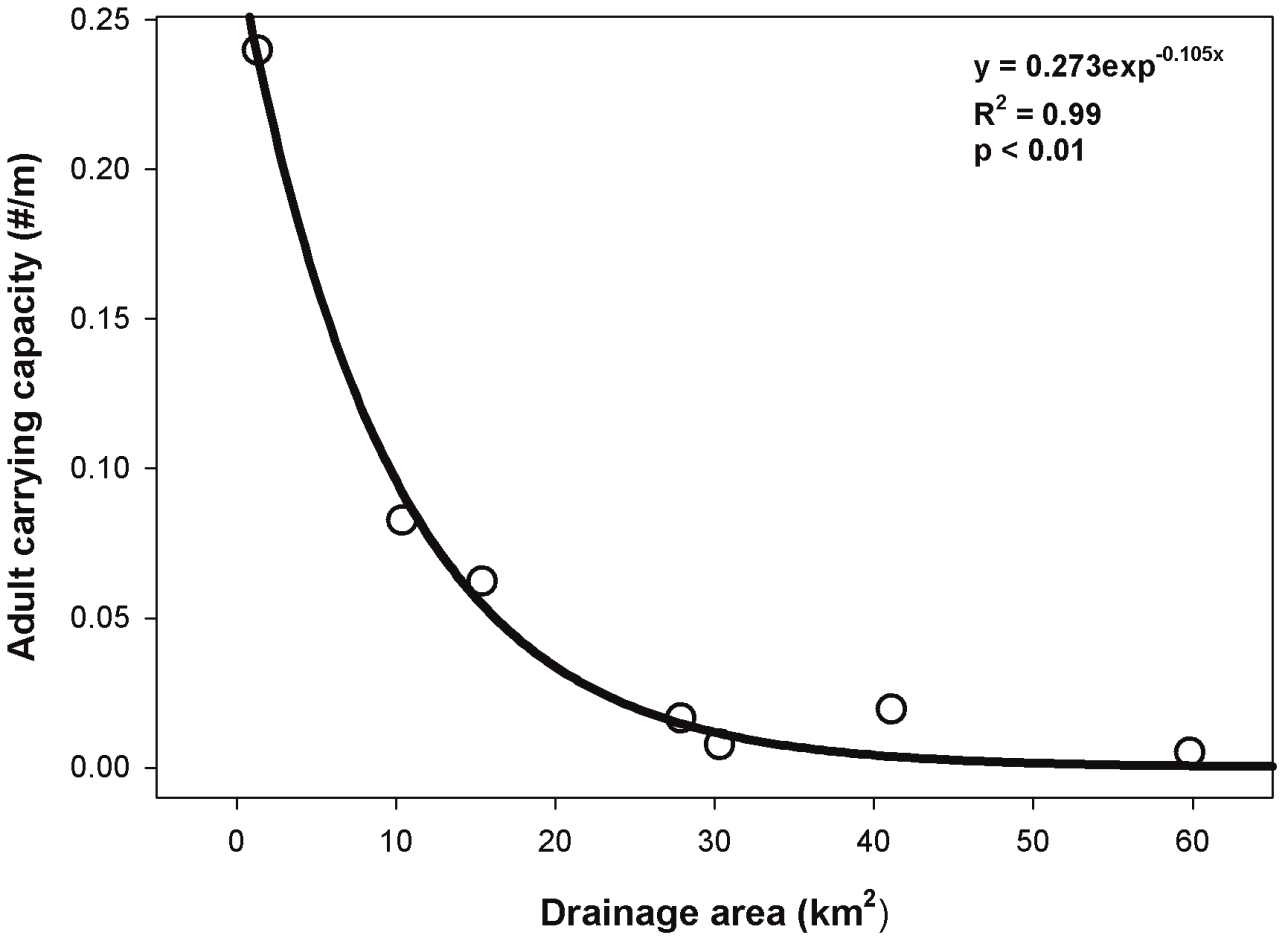
Brook trout carrying capacity along the stream continuum. Plot of the local adult carrying capacity as a function of stream drainage area (kilometers^2^). Carrying capacity was estimated as the point where radult  = 0 when plotted as a function of local adult density (see Figure 5).

When plotting adult brook trout carrying capacities against stream drainage area, a significant exponential decline in carrying capacity was observed (*p*<0.01, Figure 9). This suggests that brook trout population size amongst habitats is limited by various mechanisms along this stream continuum. Results from this study and other systems suggest that competition for food and / or foraging habitat may be the dominant density-dependent mechanism regulating brook trout populations in small headwater streams that demonstrate ideal growth temperatures for brook trout [45], [54], [55], [56]. Interestingly this is also consistent with processes known to affect mottled sculpin populations within their core distribution [14], [57], [58].

Extremely high prey productivity [28] and greater growth potential of brook trout residing in the main stem [59], [60] suggest that mechanisms other than competition for food may regulate brook trout populations in more peripheral habitats. One explanation for strong regulation of peripheral populations is based on source-sink theory [61]. Dispersal of brook trout from strongly regulated core / source populations in the headwaters may produce strong density-dependence in the peripheral / sink populations in larger main stems. Evidence of density-dependence in the periphery, consequently, may not be the result of local negative feedback, but rather is the result of a density-regulated source of immigrants. Under such a mechanism, when population size in the core habitat is low, population growth rates the following year are high. This results in an increased number of potential immigrants to the periphery and an increase in peripheral growth rates. High densities in the core then result in elevated growth rates in and dispersal to the periphery. Dispersal between patches potentially synchronize population dynamics among different habitat patches [62], [63]. The result is the appearance of density-dependent regulation in the periphery. However the actual mechanism would be density-dependence in the core/source habitat and a linkage to the periphery/sink habitat via dispersal. This mechanism can be used to explain the pattern of decreasing correlation with drainage area difference but lack of relationship with swim distance (Figure 8). If brook trout movement is from a core/source (tributary) to a sink (main stem) during years of elevated densities, then we would expect to see a stronger relationship in correlation with drainage area as opposed to swim distance. Our current analysis is insufficient to unequivocally conclude a source-sink mechanism of regulation in the periphery, but it does suggest an important avenue of further investigation within this watershed.

An alternative explanation is that larger streams may possess local carrying capacities defined by local resources. Previous research in this system suggests that brook trout inhabiting larger main stem sites may compete for thermal refugia during prolonged periods of warm and dry conditions [28]. During the spring, brook trout in the main stem may also compete for optimal growing habitat [64]. The spring is known to be an important growing period for brook trout in Shavers Fork and surrounding watersheds ([41], [59]). Competition for ideal foraging microhabitats in the main stem could then act as a potential regulating factor, where microhabitats with the greatest prey density and temperatures within optimal growth ranges would be important currency for habitat selection. Consequently, even though environmental conditions may represent a strong density-independent force in peripheral habitats, the overall dynamics of brook trout in these habitats may be predominantly influenced by density-dependent mechanisms, such as competition for suitable microhabitat.

An important factor to consider when estimating carrying capacity with this approach is harvest pressure. Unfortunately very little data exists on harvest pressure within this watershed. Neither the actual intrinsic rate of increase nor the carrying capacity should be directly affected by harvest pressure, however mortality rates and in turn densities would be. Since we observed a strong negative relationship between densities and adult intrinsic rate of increase at most sites, harvest pressure likely does not strongly influence our estimates of carrying capacity. However, if harvest pressure showed a strong direct relationship to brook trout densities, then harvest could be a strong regulating factor within this watershed. Hixon and Carr [65] found that in the absence of a resident and transient predator, the reef fish *Chromis cyanea* demonstrated density-independence. However, in the presence of both predators, the reef fish showed strong density-dependent mortality. Therefore, our estimates of carrying capacity in the main stem of this watershed could potentially be influenced by the presence of strong harvest pressure, however, lack of data on harvest pressure limits our ability to further investigate this mechanism.

Although density-independent factors were rarely the most parsimonious models in candidate sets, most density-independent models were interpretable and accounted for a substantial amount of variability when in combination with density-dependent models (Table 2). For the majority of interpretable temperature models, we observed a positive relationship between population growth rates and temperature (Table 2). In all four main stem sites, per capita growth of total and adult brook trout tended to be greatest in years with intermediate spring-time temperatures (∼12.5°C, Figures 4 and 5). This pattern is consistent with population dynamics of other salmonid species when modeled as a function of DI mechanisms. For example, Isaak & Hubert [66] observed the greatest total trout abundances (*Oncorhynchus clarki*, *Salmo trutta*, and *Salvelinus fontinalis*) at streams with temperatures of approximately 12°C, regardless of the stream size and distribution. Additionally, mortality and recruitment rates for brown trout populations in northwestern Spain have been observed to be best suited for intermediate discharge rates [5].

Within core headwater habitats, YOY recruitment was positively correlated with both spring and summer water temperature. Spawning and YOY dynamics are predominantly controlled by density-independent mechanisms, such as stream temperature and discharge [5], [38], [43], [53], [67]. However, the positive relationship between temperature and brook trout per capita growth rates that we observed across size classes suggest that elevated mortality rates in response to warmer temperatures is less likely to influence population dynamics within this watershed than temperature effects on trout dispersal [28].

Our current results, combined with previous studies, provide a good picture of the density-dependent and density-independent forces influencing brook trout populations across stream size gradients [44], [45], [53], [55], [66]–[68]. In particular, density-independent effects on YOY may actually reflect an indirect stock-recruitment relationship. Higher summer temperatures likely reduce the number of adult brook trout that emigrate from cooler headwater sites [59]. The higher number of spawning adults within headwaters would then increase the number of YOY the following year as a result of a strong positive relationship between YOY densities and adult densities the previous year (Table 3, Figure 7). Year to year variation in juvenile recruitment coupled with strong density-dependent feedback then sets adult population sizes in subsequent years. Consequently, our evidence suggests that the density-independent variables, such as water temperature, may be less important for controlling survival rates and more important for behavioral demographic rates (*e.g.* emigration and immigration). Numerous studies have shown that salmonids will relocate within streams to select microhabitats within thermal tolerance ranges [28], [69], [70]. Therefore, density-independent mechanisms within this system may be influencing brook trout distributions within the watershed, more so than affecting mortality rates. Consequently, connectivity between small cold and larger warm streams along with behavioral decisions by adult trout may control the relationship between population dynamics and water temperature.

We predicted that C.V. of brook trout density would increase with drainage area, indicating an increase in population variability with distance from the core [17]. In the headwater 1 site, variability in population density was lowest among all sites. The expected increasing variability with drainage area was also observed, until stream size exceeded approximately 35 km^2^ (Figure 3). We believe that the low C.V. in the headwater site is likely due to high trout densities and strong DD regulation. The increasing variability in the larger streams is likely due to DI effects of temperature on dispersal behavior of larger brook trout size classes. It is unclear, however, why we observed the hump-shaped pattern in population variability when considering the full extent of stream sizes sampled. The low temporal variability in density at the largest sites along this continuum was not expected. In these sites, population sizes were always low, suggesting potentially poorer brook trout habitat. There may be substantial discrepancies in the amount of “preferred” microhabitat within these main stem sites. Hansbarger et al. [71] and Petty et al. [28] showed that brook trout within both main stem and tributary reaches select for specific microhabitats (*e.g.* stream velocities, depths, temperature). Therefore, differences in preferred microhabitat in this watershed could then explain the discrepancies in density variability observed here. This may be further complicated at sites such as main stem 4, where potential refugia is in close proximity to the Second Fork tributary (Figure 1, large tributary 1 and 2, and headwater 1). Correlation analysis revealed that brook trout densities from main stem 4 were out of phase with all other similar sized sites (Figure 8a), although it seemed to be more in phase with the smaller sites in the adjacent tributary. This then suggests that not only the presence of lower quality habitat, but also the proximity to higher quality habitat may drastically reduce the potential for the lower quality habitat to be occupied, even under optimal seasonal conditions.

Furthermore, brown and rainbow trout densities are much higher in these larger main stem habitats (Tables 1). Competition with non-native trout could potentially be excluding brook trout from important microhabitats. Brown trout in particular have been shown to reduce the probability of brook trout occupying some streams [72], and almost completely replace brook trout from others [73]. Consequently, peripheral sites for brook trout within this watershed may be defined by streams with drainage areas of 10–30 km^2^, and larger streams may then fit into more of an “extra-periphery” classification. The strong positive correlation between total competitor densities and brook trout densities for the peripheral sites suggests that the same factors that influence brook trout may also affect rainbow and brown trout in these streams. Furthermore, this suggests that rainbow and brown trout may add to the density-dependent regulatory effect on brook trout populations. However, competition is likely occurring at the microhabitat scale as opposed to the reach scale, which could explain why correlation analysis did not demonstrate negative relationships between brook trout and exotic species. Limited brook trout productivity as a response to local temperatures exceeding optimal growth ranges (10-19°C, [56]) and competition with exotic salmonids for important microhabitats within the “extra-periphery” may then explain the consistently lowered brook trout densities and variability over the study period [33], [37].

It is important to acknowledge that our results are limited to only one watershed within the state of West Virginia, and only one core site within this watershed. Data collection within this site was originally designed to investigate population dynamics along a continuum of drainage area rather than discrete core-periphery categories. Continued investigation within this watershed has allowed us to better understand the spatial arrangement of the brook trout population within this watershed [27], [28], [71], [74]–[76]. Although this 1 core site may not represent the typical core within this watershed, investigation of other tributaries within this watershed has led us to believe that this is a typical core tributary. Previous studies within different tributaries of the Shavers Fork watershed have shown that most productivity occurs in these small tributaries, and that the majority of brook trout within these small tributaries possess relatively sedentary life styles [27], [28]. Additionally, others have found strong synchrony in brook trout populations at the state scale [53]. Short term analyses of different headwater tributary sites in this watershed have also revealed strong synchrony among different core sites (B. M. Huntsman *unpublished manuscript*). Because of these factors, we feel confident that the small tributary used in this study represents a typical core site within the Shavers Fork watershed.

### Management implications

The prevalence of DD within this watershed suggests that any efforts to supplement brook trout productivity would require increasing the carrying capacity within the entire watershed. This would involve identifying the limiting factors setting carrying capacities in both tributaries and main stem sites. We believe that the tributaries within this watershed are strongly limited by food availability, as has been suggested for many other headwater brook trout populations [28], [54], [55], [59]. This means that increasing the amount of food as well as increasing the number of available spawning habitats (*e.g.* treating acidity, [74], [77]) would be important at the core. In more peripheral sites however, the abundance of food suggests that brook trout in the main stem are more limited in available microhabitat rather than food [28]. Consequently, maintenance of riparian cover and creation of deep coldwater refugia are essential to supplement brook trout productivity in larger main stem habitats of this watershed [75]. Additionally, reducing competition with exotic salmonids (*i.e.* rainbow and brown trout) for these limited microhabitats would also be important to enhance productivity.

Under current climate change scenarios, there are at least 2 major concerns about the response of brook trout population dynamics to increasing temperatures. First, there may be fewer “cool” years where trout will be able to disperse from headwater tributaries into main stem habitat [59]. Although this would likely have limited impact on headwater carrying capacities, it could potentially affect the total productivity within the entire watershed. With fewer brook trout moving into the main stem, there would be fewer fish supplementing their growth with main stem forage. Second, increasing temperatures would likely reduce the number of thermal refugia available in the main stem. This would then directly reduce the carrying capacity of main stem habitat, possibly to a point where it is no longer functionally available to brook trout within the watershed. This affect may be particularly acute when exotic competitors are present. Understanding how changing climate may affect the watershed scale dynamics of brook trout in this region is a priority for future research.

## Supporting Information

Table S1 ***A priori*** **models explaining response variables of brook trout time series data at different study sites.** Abbreviations are as follows: rpop  =  per capita growth rate (r = ln(n_t_/n_t−1_)) for the total brook trout population, radult  = r for adults, ryoy  =  r for young-of-the-year, dtrout  =  density of all brook trout, dadult  =  density of adult brook trout, dyoy  =  density of young-of-the-year brook trout, sp_t_T  =  mean April-June maximum temperature, su_t−1_T  =  mean July maximum temperature, and sp_t_Q  =  mean March-June discharge. The * indicates that the response variable was not analyzed in the main stem.
(DOCX)

Table S2 **All results from candidate models using AIC_c_ for 7 sites.** The first value represents the Akaike's weight (*w_i_*) given to each model in the candidate set followed by the direction of the relationship and R^2^ statistic. Bold values represent the best model in each candidate set. Both response and predictor variables follow the same notation as that in Table 2. Missing values represent predictor variables that were correlated with another predictor variable in the candidate set and therefore removed. No models were constructed for ryoy at main stem sites because few YOY were found in those sites. Models with an * were considered interpretable models using criteria from Grossman et al. [14].
(DOCX)
