# Supplementary material for: Density-Dependent Regulation of Brook Trout Population Dynamics along a Core-Periphery Distribution Gradient in a Central Appalachian Watershed
Source: PLoS One. 2014 Mar 11;9(3):e91673. doi: 10.1371/journal.pone.0091673 (PMC3950256; doi:10.1371/journal.pone.0091673)
Supplement: Table S1 — A priori models explaining response variables of brook trout time series data at different study sites. Abbreviations are as follows: rpop = per capita growth rate (r = ln(nt/nt−1)) for the total brook trout population, radult = r for adults, ryoy = r for young-of-the-year, dtrout = density of all brook trout, dadult = density of adult brook trout, dyoy = density of young-of-the-year brook trout, sptT = mean April-June maximum temperature, sut−1T = mean July maximum temperature, and sptQ = mean March-June discharge. The * indicates that the response variable was not analyzed in the main stem. (DOCX) [file pone.0091673.s001.docx]

**Table S1: *A priori* models explaining response variables of brook trout time series data at different study sites.**

| **Location** | **Explanatory Mechanism** | **Statistical Hypotheses** | **Response Variable** | **Candidate Models** |
| --- | --- | --- | --- | --- |
| Headwater, Large Tributary, Main stem | Simple density-dependence (DD): interspecific competition for thermal refuge and food limits response variables | Negative relationship between response variables and densities of trout | rpop, radult, ryoy* | -dtrout_t-1_, -dadult_t-1_, -dyoy_t-1_ |
| Headwater, Large Tributary, Main stem | Simple recruitment limitation (RL): YOY densities at t-1 limits adult growth rates, and adult densities at t-1 limits YOY growth rates and densities | Positive relationship between response variables and densities of trout | rpop, radult, ryoy*, | dadult_t-1_, dyoy_t-1_ |
| Headwater, Large Tributary | Simple density-independence (DI): **1)** decreased emigration rates during elevated spring growth temperatures, thermal refuge during harsh summer temperatures, and elevated mortality during high flow events limit response variables | Positive relationship between response variables and both temperature variables. Negative relationship between response variables and flow | rpop, radult, ryoy | sp_t_T, su_t-1_T, -sp_t_Q |
| Headwater, Large Tributary | **2)** Elevated survival and prey delivery during high flow events limit response variables | Positive relationship between response variables and flow | rpop, radult, ryoy | sp_t_Q |
| Main stem | Simple density-independence (DI): optimal growth conditions during elevated spring growth temperatures, increased emigration due to limited thermal refuge during harsh summer temperatures, and elevated mortality during high flow events limit response variables | Positive relationship between response variables and growth temperature. Negative relationship between response variables and both summer temperatures and flow | rpop, radult | sp_t_T, -su_t-1_T, - sp_t_Q |
| Headwater, Large Tributary | Complex density-independence (CDI): **1)** high survival rates due to optimal growth temperature and flow conditions limit response variables | Positive relationship between response variables and both growth temperature and flow | rpop, radult, ryoy | sp_t_T, sp_t_Q |
| Headwater, Large Tributary, Main stem | **2)** high survival rates due to optimal growth temperature and high mortality rates during high flow conditions limit response variables | Positive relationship between growth temperature and response variables and negative relationship between response variables and flow | rpop, radult | sp_t_T, - sp_t_Q |
| Headwater, Large Tributary, Main stem | Complex multi-mechanism models (MM): multiple DD, DI, and RL mechanisms limit response variables | Negative or Positive relationships between response variables and multiple DD, DI, and RL effects | rpop, radult, ryoy* | dtrout_t-1_, dadult_t-1_, dyoy_t-1_, sp_t_T, su_t-1_T, sp_t_Q |
| Headwater, Large Tributary, Main stem | Global: DD, RL, and DI |  | rpop, radult, ryoy* | dtrout_t-1_, dadult_t-1_, dyoy_t-1_, sp_t_T, su_t-1_T, sp_t_Q |

Abbreviations are as follows: rpop = per capita growth rate (r = ln(n_t_/n_t-1_)) for the total brook trout population, radult = r for adults, ryoy = r for young-of-the-year, dtrout = density of all brook trout, dadult = density of adult brook trout, dyoy = density of young-of-the-year brook trout, sp_t_T = mean April-June maximum temperature, su_t-1_T = mean July maximum temperature, and sp_t_Q = mean March-June discharge. The * indicates that the response variable was not analyzed in the main stem.
