# Supplementary material for: Density-Dependent Regulation of Brook Trout Population Dynamics along a Core-Periphery Distribution Gradient in a Central Appalachian Watershed
Source: PLoS One. 2014 Mar 11;9(3):e91673. doi: 10.1371/journal.pone.0091673 (PMC3950256; doi:10.1371/journal.pone.0091673)
Supplement: Table S2 — All results from candidate models using AICc for 7 sites. The first value represents the Akaike's weight (wi) given to each model in the candidate set followed by the direction of the relationship and R2 statistic. Bold values represent the best model in each candidate set. Both response and predictor variables follow the same notation as that in Table 2. Missing values represent predictor variables that were correlated with another predictor variable in the candidate set and therefore removed. No models were constructed for ryoy at main stem sites because few YOY were found in those sites. Models with an * were considered interpretable models using criteria from Grossman et al. [14]. (DOCX) [file pone.0091673.s002.docx]

**Table S2: All results from candidate models using AIC_c_ for 7 sites.**

| **Response** | **Predictor** | **HEADWATER** | **LARGE TRIBUTARY** | | **MAIN STEM** | | | |
| --- | --- | --- | --- | --- | --- | --- | --- | --- |
| **Variable** | **Variable** | **1** | **1** | **2** | **1** | **2** | **3** | **4** |
| rpop | dtrout_t-1_ | 0.225 (-0.28)* | **0.491 (-0.29)*** | **0.465 (-0.41)*** | **0.709 (-0.46)*** | 0.253 (-0.16)* | **0.588 (-0.38)*** | **0.710 (-0.50)*** |
|  | dyoy_t-1_ | 0.069 (-0.07)* |  | 0.156 (-0.25)* |  |  | 0.072 (-0.01)* |  |
|  | su_t-1_T | 0.074 (+0.08)* | 0.139 (+0.06)* | 0.043 (-0.00) | 0.068 (-0.09) | 0.122 (-0.01)* |  | 0.031 (+0.00) |
|  | sp_t_T | 0.050 (-0.00)* | 0.109 (+0.01)* | 0.112 (-0.19)* | 0.077 (+0.12)* | **0.464 (+0.27)*** | 0.213 (+0.22)* | 0.041 (-0.07) |
|  | sp_t_Q | 0.110 (+0.16)* | 0.108 (+0.01)* | 0.044 (+0.00) | 0.054 (-0.04) | 0.118 (+0.01)* | 0.074 (-0.02)* | 0.049 (+0.10) |
|  | sp_t_T+sp_t_Q | 0.003 (-/+0.19) | 0.004 (+/+0.01) | 0.004 (-/+0.23) | 0.003 (+/-0.16) | 0.014 (+/+0.27) | 0.006 (+/-0.24) | 0.002 (-/+0.18) |
|  | su_t-1_T+dtrout_t-1_ | **0.450 (+/-0.72)*** | 0.123 (+/-0.57)* | 0.016 (+/-0.44) | 0.025 (+/-0.49) | 0.007 (+/-0.17) |  | 0.042 (+/-0.58) |
|  | sp_t_T+dtrout_t-1_ | 0.006 (-/-0.29) | 0.013 (-/-0.29) | 0.133 (-/-0.65)* | 0.035 (+/-0.53) | 0.017 (+/-0.31) | 0.021 (+/-0.42) | 0.106 (-/-0.66)* |
|  | sp_t_Q+dtrout_t-1_ | 0.020 (+/-0.37) | 0.014 (-/-0.30) | 0.013 (+/-0.41) | 0.029 (-/-0.51) | 0.007 (+/-0.16) | 0.017 (-/-0.39) | 0.020 (-/-0.51) |
|  | su_t-1_T+dyoy_t-1_ | 0.003 (+/-0.16) |  | 0.004 (-/-0.25) |  |  |  |  |
|  | sp_t_T+dyoy_t-1_ | 0.002 (+/-0.07) |  | 0.006 (-/-0.29) |  |  | 0.006 (+/-0.22) |  |
|  | sp_t_Q+dyoy_t-1_ | 0.004 (+/-0.23) |  | 0.004 (-/-0.25) |  |  | 0.002 (-/-0.07) |  |
|  | global | 0.000 (0.87) | 0.000 (0.60) | 0.000 (0.73) | 0.000 (0.60) | 0.000 (0.32) | 0.000 (0.62) | 0.000 (0.82) |
| radult | dadult_t-1_ | **0.240 (-0.17)*** | **0.456 (-0.22)*** | **0.289 (-0.28)*** | **0.766 (-0.51)*** | 0.364 (-0.26)* | **0.670 (-0.43)*** | **0.707 (-0.55)*** |
|  | dyoy_t-1_ | 0.143 (-0.07)* |  | 0.236 (-0.25)* |  |  | 0.056 (-0.01) | 0.038 (-0.14) |
|  | su_t-1_T | 0.153 (+0.08)* | 0.193 (+0.06)* | 0.066 (-0.00)* | 0.046 (-0.09) | 0.102 (-0.01)* |  | 0.019 (+0.00) |
|  | sp_t_T | 0.104 (-0.00)* | 0.152 (+0.01)* | 0.170 (-0.19)* | 0.052 (+0.12) | **0.385 (+0.27)*** | 0.164 (+0.22)* | 0.026 (-0.07) |
|  | sp_t_Q | 0.228 (+0.16)* | 0.151 (+0.01)* | 0.066 (+0.00)* | 0.037 (-0.04) | 0.098 (+0.01)* | 0.057 (-0.02) | 0.031 (0.10) |
|  | sp_t_T+sp_t_Q | 0.007 (-/+0.19) | 0.004 (+/+0.01) | 0.006 (-/+0.23) | 0.002 (+/-0.16) | 0.011 (+/+0.27) | 0.005 (+/-0.24) | 0.001 (-/+0.18) |
|  | su_t-1_T+dadult_t-1_ | 0.087 (+/-0.53)* | 0.016 (+/-0.27) | 0.009 (+/-0.31) | 0.027 (+/-0.54) | 0.010 (+/-0.26) |  | 0.032 (+/-0.60) |
|  | sp_t_T+dadult_t-1_ | 0.007 (-/-0.19) | 0.013 (+/-0.23) | 0.128 (-/-0.61)* | 0.047 (+/-0.59) | 0.020 (+/-0.36) | 0.022 (+/-0.46) | 0.121 (-/-0.70)* |
|  | sp_t_Q+dadult_t-1_ | 0.011 (+/-0.27) | 0.015 (+/-0.26) | 0.008 (+/-0.29) | 0.025 (-/-0.53) | 0.010 (+/-0.26) | 0.019 (-/-0.43) | 0.020 (-/-0.55) |
|  | su_t-1_T+dyoy_t-1_ | 0.006 (+/-0.16) |  | 0.006 (-/-0.25) |  |  |  | 0.001 (+/-0.20) |
|  | sp_t_T+dyoy_t-1_ | 0.004 (+/-0.07) |  | 0.009 (-/-0.29) |  |  | 0.004 (+/-0.22) | 0.002 (-/-0.25) |
|  | sp_t_Q+dyoy_t-1_ | 0.009 (+/-0.23) |  | 0.006 (-/-0.25) |  |  | 0.002 (-/-0.07) | 0.001 (+/-0.18) |
|  | global | 0.000 (0.87) | 0.000 (0.38) | 0.000 (0.73) | 0.000 (0.64) | 0.000 (0.37) | 0.000 (0.62) | 0.000 (0.82) |
| ryoy | dadult_t-1_ | 0.093 (+0.12)* | 0.215 (-0.07)* | 0.005 (+0.07) |  |  |  |  |
|  | dyoy_t-1_ | **0.306 (-0.32)*** | **0.409 (-0.19)*** | **0.800 (-0.69)*** |  |  |  |  |
|  | su_t-1_T | 0.212 (+0.27)* |  | 0.007 (+0.11) |  |  |  |  |
|  | sp_t_T | 0.056 (-0.01)* | 0.159 (-0.00)* | 0.015 (-0.26) |  |  |  |  |
|  | sp_t_Q | 0.149 (+0.21)* | 0.168 (+0.01)* | 0.005 (-0.01) |  |  |  |  |
|  | sp_t_T+sp_t_Q | 0.006 (-/+0.28) | 0.005 (-/+0.02) | 0.000 (-/+0.27) |  |  |  |  |
|  | su_t-1_T+dadult_t-1_ | 0.006 (+/+0.27) |  | 0.000 (+/+0.14) |  |  |  |  |
|  | sp_t_T+dadult_t-1_ | 0.003 (-/+0.12) | 0.006 (-/-0.07) | 0.000 (-/+0.28) |  |  |  |  |
|  | sp_t_Q+dadult_t-1_ | 0.016 (+/+0.42) | 0.007 (+/-0.09) | 0.000 (-/+0.08) |  |  |  |  |
|  | su_t-1_T+dyoy_t-1_ | 0.102 (+/-0.61)* |  | 0.100 (+/-0.78)* |  |  |  |  |
|  | sp_t_T+dyoy_t-1_ | 0.008 (+/-0.32) | 0.013 (-/-0.22) | 0.025 (-/-0.70) |  |  |  |  |
|  | sp_t_Q+dyoy_t-1_ | 0.041 (+/-0.52)* | 0.018 (-/-0.27) | 0.042 (-/-0.73) |  |  |  |  |
|  | global | 0.000 (0.93) | 0.000 (0.29) | 0.000 (0.88) |  |  |  |  |
|  |  |  |  |  |  |  |  |  |

The first value represents the Akaike’s weight (*w_i_*) given to each model in the candidate set followed by the direction of the relationship and R^2^ statistic. Bold values represent the best model in each candidate set. Both response and predictor variables follow the same notation as that in Table 2. Missing values represent predictor variables that were correlated with another predictor variable in the candidate set and therefore removed. No models were constructed for ryoy at main stem sites because few YOY were found in those sites. Models with an * were considered interpretable models using criteria from Grossman et al. [14].
